# Pulp or Peel? Comparative Analysis of the Phytochemical Content and Selected Cosmetic-Related Properties of *Annona cherimola* L., *Diospyros kaki* Thumb., *Cydonia oblonga* Mill. and *Fortunella margarita* Swingle Pulp and Peel Extracts

**DOI:** 10.3390/molecules29051133

**Published:** 2024-03-03

**Authors:** Magdalena Lasota, Paulina Lechwar, Wirginia Kukula-Koch, Marcin Czop, Karolina Czech, Katarzyna Gaweł-Bęben

**Affiliations:** 1 Department of Cosmetology, University of Information Technology and Management in Rzeszów, Sucharskiego 2, 35-225 Rzeszów, Poland; mlasota@wsiz.edu.pl (M.L.); plechwar@wsiz.edu.pl (P.L.);; 2 Department of Pharmacognosy, Medical University of Lublin, Chodźki 1, 20-093 Lublin, Poland; virginia.kukula@gmail.com; 3 Department of Clinical Genetics, Medical University of Lublin, Radziwiłłowska 11, 20-080 Lublin, Poland; marcin.czop@umlub.pl

**Keywords:** *Annona cherimola*, *Diospyros kaki*, *Cydonia oblonga*, *Fortunella margarita*, antioxidant, tyrosinase, keratinocytes, fibroblasts, sun protection factor, HPLC-MS

## Abstract

Fruit peels might be a valuable source of active ingredients for cosmetics, leading to more sustainable usage of plant by-products. The aim of the study was to evaluate the phytochemical content and selected biological properties of hydroglycolic extracts from peels and pulps of *Annona cherimola*, *Diospyros kaki*, *Cydonia oblonga*, and *Fortunella margarita* as potential cosmetic ingredients. Peel and pulp extracts were compared for their antiradical activity (using DPPH and ABTS radical scavenging assays), skin-lightening potential (tyrosinase inhibitory assay), sun protection factor (SPF), and cytotoxicity toward human fibroblast, keratinocyte, and melanoma cell lines. The total content of polyphenols and/or flavonoids was significantly higher in peel than in pulp extracts, and the composition of particular active compounds was also markedly different. The HPLC-MS fingerprinting revealed the presence of catechin, epicatechin and rutoside in the peel of *D. kaki*, whereas kaempferol glucoside and procyanidin A were present only in the pulp. In *A. cherimola*, catechin, epicatechin and rutoside were identified only in the peel of the fruit, whereas procyanidins were traced only in the pulp extracts. Quercetin and luteolinidin were found to be characteristic compounds of *F. margarita* peel extract. Naringenin and hesperidin were found only in the pulp of *F. margarita*. The most significant compositional variety between the peel and pulp extracts was observed for *C. oblonga*: Peel extracts contained a higher number of active components (e.g., vicenin-2, kaempferol rutinoside, or kaempferol galactoside) than pulp extract. The radical scavenging potential of peel extracts was higher than of the pulp extracts. *D. kaki* and *F. margarita* peel and pulp extracts inhibited mushroom and murine tyrosinases at comparable levels. The *C. oblonga* pulp extract was a more potent mushroom tyrosinase inhibitor than the peel extract. Peel extract of *A. cherimola* inhibited mushroom tyrosinase but activated the murine enzyme. *F. margarita* pulp and peel extracts showed the highest in vitro SPF. *A. cherimola*, *D. kaki*, and *F. margarita* extracts were not cytotoxic for fibroblasts and keratinocytes up to a concentration of 2% (*v/v*) and the peel extracts were cytotoxic for A375 melanoma cells. To summarize, peel extracts from all analyzed fruit showed comparable or better cosmetic-related properties than pulp extracts and might be considered multifunctional active ingredients of skin lightening, anti-aging, and protective cosmetics.

## 1. Introduction

Edible fruits are known as rich sources of nutrients (carbohydrates, vitamins, and minerals) as well as non-nutrients, especially polyphenols, known for their various health-promoting properties [1]. Due to increased knowledge regarding the health benefits of fresh fruit consumption, the global production of fruits constantly increases, reaching over 909.6 million tones in 2021 [2]. Increased fruit consumption also results in the generation of higher amounts of fruit-related waste and by-products, such as peels, seeds, and pomace. Several studies showed that these parts of fruits are still rich sources of biologically active phytochemicals, including polyphenolic compounds, and thus might be used as an easily available source of active ingredients for dietary supplements and cosmetics [3]. In respect of some fruit species, the phytochemical content of inedible parts such as peels is more complex than the composition of edible pulp, and the extracts obtained from edible and inedible compartments may differ significantly in their biological properties. For example, the concentration of phenolics in extracts from the peels of lemons, grapes, and oranges is ca. 15% higher than in pulp extracts from the same fruits [4,5]. Comparative analysis of the bioactive properties and phytochemical content of extracts obtained from edible and nonedible parts of common fruits is important for sustainable utilization of natural resources and reducing the amount of unused or wasted biomass. The valorization of fruit waste will help reduce environmental pollution and lead to value-added products [6]. One of the possible applications of bioactive compounds obtained from fruit by-products and nonedible parts is their application in cosmetic products. The global cosmetics market is emerging as one of the fastest-growing industries of the past decade [7]. Growing consumer awareness of the impact of cosmetic ingredients on health and the environment triggers an increased demand for care products based on natural ingredients [8]. Obtaining bioactive molecules from fruit by-products and nonedible parts may therefore be a solution to the growing demand in the cosmetics market for natural substances [3]. In consideration of the above, the aim of this study was to evaluate the phytochemical content and selected, cosmetic-related properties of hydroglycolic extracts obtained from the pulp (edible) and peels (nonedible) of four types of fruit: Cherimoya (*Annona cherimola* Miller), persimmon (*Diospyros kaki* Thumb.), quince (*Cydonia oblonga* Mill.), and kumquat (*Fortunella margarita* (Loureiro) Swingle) to prove their suitability as cosmetic ingredients. The four fruits were selected for the studies based on some previously published data indicating that the peels of these fruits are rich in active compounds with antioxidant activity [9,10,11,12,13,14,15]. However, to our best knowledge, the sun-protecting potential, tyrosinase inhibitory activity, and cytotoxicity against human skin cells (cancer and non-cancerous) have not been compared to date between peel and pulp extracts of selected fruits.

Cherimoya is a small subtropical tree from the Annonaceae family that produces edible fruit with a delicate, rich, and aromatic flavor. Cherimoya has been cultivated since the times of the Incan Empire, and in the last few decades, it has become endemic in several subtropical areas of the world. In Mexican traditional medicine, cherimoya has been used to treat inflammation, cough, headache, fever, diabetes, and parasites, either alone or in combination with other herbal medicines [16]. Several recent studies showed that different parts of cherimoya (especially fruit and leaves) possess an interesting phytochemical profile, are rich in polyphenols and alkaloids [17], and are valuable sources of active ingredients for traditional medicine preparation for the treatment of gastric, intestinal, cardiovascular, skin, and eye diseases [18]. According to recent research data, the extracts and active compounds obtained from *A. cherimola* possess antibacterial, antiviral, antidiabetic, anti-hyperlipidemic, and anticancer properties [19,20]. In modern cosmetics, Annona Cherimola Fruit Extract is used in cosmetic products as a skin-conditioning ingredient, and hydrolyzed Annona Cherimola fruit extract is applied as a skin-conditioning and antioxidant component of cosmetic formulations [21]. Peel extracts from cherimoya fruit are not currently used in cosmetics, but some recent data indicate their potentially interesting cosmetic-related properties [22].

Persimmon, belonging to the Ebenaceae family, is a very popular fruit in East Asian countries. Recently, persimmon’s popularity has grown outside its traditional region of production (Korea, China, and Japan), growing into an encouraging crop in Brazil and Mediterranean countries such as Spain, Portugal, and Italy [23]. The world production of persimmon is around five million tons, corresponding to 0.75% of total fruit production. [2] In addition to its nutritional value, persimmon fruit is also an important ingredient used in traditional Chinese medicine for its beneficial effects on health, including maintaining body temperature, improving the function of the lungs, stomach, spleen, and intestines, and preventing and treating hypertension, diabetes, atherosclerosis, insomnia, thrush, and sore throat. The health benefits of persimmon fruit have been associated with its richness in bioactive metabolites, including phenolic compounds, carotenoids, and vitamins [24]. In several studies, it has been described that the concentration of nutrients and other functional components of persimmon is higher in the skin than in the pulp [11]. However, the peel of the fruit is typically discarded following the consumption of ripe fruit or during the fruit drying process—a common method for persimmon preservation in Asian countries [25]. In cosmetics, ingredients obtained from the whole fruit or peels of persimmon can be found. Diospyros Kaki Fruit Extract, Peel Extract, and Peel Water (steam distillate) are used as skin-conditioning components; Diospyros Kaki Fruit Powder is also known as a deodorant and emollient; and Diospyros Kaki Fruit Juice is registered as an antioxidant, astringent, deodorant, and oral care cosmetic ingredient [21].

Quince belongs to the *Maloideae* subfamily of the Rosaceae family, including several commercially important fruits such as pears and apples. Because of the hardness, acidity, and astringency of raw quince fruit, it is not edible unprocessed. Nevertheless, it is often used for the production of jams, jellys, liqueurs, and marmalades. Quince is also used in canning and for aromatic distillation. *Cydonia oblonga* is a native fruit of Iran, but it is also now cultivated around the world. It is known in Iranian traditional medicine as a remedy for a variety of diseases. Mucilaginous mass obtained by soaking quince seeds in water has been used for wound healing, reducing scars, and as an anti-wrinkle treatment. The wound healing, anti-inflammatory, and anti-allergic potential of quince extracts has also been supported by modern research [26]. In the Cosmetic Ingredient Database (CosIng), only two ingredients obtained from *C. oblonga* can be found—Cydonia Oblonga Leaf Extract and Cydonia Oblonga Fruit Water (steam distillate from the whole fruit), both with skin-conditioning properties [21]. The effectiveness of quince extracts in skin care has been recently confirmed by Khilje et al., who investigated the cosmetic properties of an emulgel containing 4% of fresh quince fruit extracts on human volunteers. Following the three-month application, the formulation reduced sebum and erythema levels, while the elasticity and moisture content showed increments [27].

Kumquat is a small citrus tree native to southeastern China and cultivated in several parts of the globe, such as Japan, India, Australia, the USA, Brazil, and Europe. Kumquat fruits, the smallest among all citrus fruits, are rich in nutrients and bioactive compounds, including flavonoids, phenolic acids, vitamins, and amino acids [28]. *F. margarita* has been used in attempts to prevent the rupture of blood vessels, reduce the fragility and permeability of blood capillaries, and slow the hardening of arteries. It is used in traditional herbal medicines, especially for the treatment of coughs and colds [29]. The peel of kumquat fruit is edible, but it is often separated from the fruit pulp and used for the extraction of essential oil [15]. In cosmetics, Fortunella Margarita Fruit Extract and Fruit Water (steam distillate) are used as skin-conditioning ingredients, and Fortunella Margarita Peel Oil (volatile) is used as a fragrance and flavoring component of the formulation [21].

Extracts obtained from the mentioned fruit species are currently listed as cosmetic ingredients and are thus considered safe for the skin. However, despite some scientific evidence that peels are richer in bioactive compounds than other parts of these fruits, there is no comparative evaluation of their phytochemical composition and biological activities in respect of replacing the whole fruit extracts in cosmetics with extracts from the peels, which are currently considered wastes rather than the source of valuable cosmetics ingredients.

## 2. Results and Discussion

### 2.1. Comparative Analysis of Total Phenolic, Flavonoid and Protein Contents between Peel and Pulp Hydroglycolic Extracts

Effective use of fruit by-products is possible when the method of their further processing is simple and cost effective. Therefore, in this study, we proposed ultrasonic-assisted extraction (UAE) in a water:propylene glycol mixture (4:1, *v:v*) as an easy and green extraction procedure for obtaining active ingredients for cosmetic use. Hydroglycolic extracts, following proper filtration, might be directly applied in various types of cosmetic formulations (e.g., emulsion, tonic, washing cosmetics). Propylene glycol (PG) is known as a humectant and a promoter of the penetration of active substances through the epidermis. PG can be obtained from plant sources and is accepted in natural and vegan cosmetics. Moreover, it is readily biodegradable under aerobic conditions in freshwater, seawater, and soil, and thus is not considered an environmental hazard, in contrast with several other organic solvents [30]. Our previous studies showed that the addition of PG to water during the extraction procedure might significantly increase the content of bioactive compounds as well as the final activity of the obtained extract [31,32].

Hydroglycolic peel and pulp extracts were compared for their total polyphenolic content (TPC), total flavonoid content (TFC), and protein content, as summarized in Table 1. In respect of *A. cherimola*, *D. kaki*, and *C. oblonga* the TPC in the peel extracts was significantly higher than in the pulp extracts. For *A. cherimola*, the TPC in the peel extract was almost 4.5 times higher than in the pulp. The peel of *A. cherimola* also contained ca. 30 times more flavonoids than the pulp. The TPC of *F. margarita* extracts from peel and pulp was comparable; however, the peel extract contained about 3.7 times more flavonoids than the pulp. Detectable amounts of protein were found only in the peel extract of *D. kaki* (no protein detected in the pulp extract) and the pulp and peel extracts of *C. oblonga* (no significant difference between pulp and peel).

The content of bioactive compounds in hydroglycolic extracts from analyzed fruits has not been studied to date. Therefore, the obtained values might be compared only with the literature data regarding the alcoholic or hydroalcoholic extracts described previously. In general, usage of ethanol or methanol alone or in mixture with water also resulted in higher extraction of polyphenols and flavonoids from the peels of the mentioned fruits [9,10,11,12,13,14,15], which is comparable with the data regarding the hydroglycolic extracts.

Loizzo et al. compared TPC and TFC between the peel and pulp extracts from fresh *A. cherimola* fruit. In this analysis, peel extract contained slightly more polyphenols than pulp extract (14.6 ± 1.1 versus 12.6 ± 0.8 mg chlorogenic acid equivalents/100 g fresh weight, respectively) and significantly more flavonoids (8.2 ± 1.2 versus 3.8 ± 0.6 mg QE/100g fresh weight, respectively) [9]. Higher TPC in the peel than pulp extracts of two *A. cherimola* cultivars grown in Spain was reported by García-Salas and co-workers [10]. Hydroethanolic extracts obtained from the peels of various cherimoya cultivars collected in Portugal showed higher TPC and TFC than the extracts from the pulps and seeds of the same fruits. The TPC of the analyzed extracts varied from 17.0–19.6 mg GAE/100 g of fresh fruit, and the TFC was from 33.0–44.7 mg ECE/100 g. Total protein content in *A. cherimola* peel extracts was from 1.36–1.96 g/100 g [18]. A slightly higher TPC (28.771 ± 0.008 mg GAE/g dried extract) was detected in 80% ethanol peel extract from the *A. cherimola* “Fino de Jete” variety [22].

Lee et al. compared TPC and TFC in persimmon juice obtained from cultivars grown in different regions of Korea. TPC in the fruit juice ranged from 70.44 to 298.01 mg GAE/100 g, and TFC varied from total flavonoid contents in persimmon fruit juice from different regions, ranging from 7.57 to 31.22 mg rutin equivalents/100 g [33]. TPC in *D. kaki* whole fruits collected in 6 locations in Italy varied from 45–186 mg/kg of fresh fruit weight [34]. A higher TPC in the peel than the pulp of *D. kaki* was reported for the first time by Gorinstein and co-workers [11]. In another study, Jeong et al. compared the TPC and TFC of *D. kaki* peel extracts in various solvents (n-hexane, chloroform, ethyl acetate, n-butanol, and water). The highest values of TPC and TFC were found for ethyl acetate extracts (17,242.9 ± 2096.4 mg GAE/100 g dried weight and 5616.0 ± 728.2 mg QE/100 g dried weight, respectively). Aqueous extract contained the lowest TPC (402.1 ± 38.1 mg GAE/100 g), and chloroform extract showed the lowest TFC (9.0 ± 12.3 mg QE/100 g) [12]. The content of bioactive compounds in both the peel and pulp of persimmon fruit also changes during ripening, reaching its highest levels at the fully mature stage [35].

Hydroglycolic extracts from quince were found to contain the highest amounts of total polyphenols among all analyzed extracts, which is in agreement with previously published data presenting quince fruit as a particularly rich source of polyphenols; however, the hydroglycolic extracts from quince were not studied to date. Khiljee et al. showed that a methanolic extract prepared from fresh whole fruits of *C. oblonga* contained 25.48 ± 1.52 mg GAE/g DW polyphenols and 14.76 ± 0.84 QE/g DW flavonoids [27]. In another study, methanolic extracts from frozen whole fruits of *C. oblonga* contained even higher amounts of polyphenols and flavonoids, accounting for 158.73 ± 8.74 mg GAE/g DW and 27.34 ± 1.40 mg QE/g DW, respectively [36]. Quince peel has been reported in several studies to contain higher levels of active compounds compared to the pulp and seed [13]. In the study by Fattouch et al., the TPC of the acetone/water (3:1, *v/v*) extracts from the peel, pulp, and seeds of *C. oblonga* ranged from 105 to 157 and 37 to 47 mg/g in peel and pulp, respectively [14]. In another study, the TPCs of methanolic extracts from quince fruit parts were found to be 6.3, 2.5, and 0.4 g/kg for peel, pulp, and seeds, respectively [37]. Hanan et al. compared TPC and TFC in four extracts from quince peel. The lowest TPC and TFC was measured in the aqueous extract (14.41 ± 1.00 mg GAE/g DW and 8.7 ± 0.71 mg RE/g DW, respectively), and the highest in the hydroethanolic extract (27.23 ± 0.85 mg GAE/g DW and 16.5 ± 1.02 mg RE/g DW, respectively) [38]. Szychowski et al. showed that both lipophilic and hydrophilic extracts of quince peel have higher phenolic contents with greater antioxidant activity. The peel-to-pulp TPC ratio (TPC peel/TPC pulp) was about 4.7 [39].

To our best knowledge, the TPC and TFC of kumquat peel and pulp have not been directly compared in the scientific literature. It is, however, known that whole fruit extract contains significant amounts of polyphenols, and the TPC depends on the type of solvent used for extraction. According to Jayaprakasha et al., the ethyl acetate extract from the whole fruit contained the highest TPC (170 ± 19 mg catechin equivalents (CE)/g of extract), and the lowest TPC (79 ± 9.1 mg CE/g) was measured in 75% MetOH [40]. Sadek and co-workers compared TPC in the ethyl acetate, dichloromethane, and butanol fractions of peel extracts obtained from Greek and Egyptian persimmon cultivars, showing the highest TPC in the ethyl acetate fraction (77.4 ± 0.2 and 106.2 ± 0.5 mg GAE/g dry fraction for Greek and Egyptian cultivars, respectively) [15].

### 2.2. HPLC-ESI-QTOF-MS/MS Fingerprinting of the Analyzed Extracts

The introduced conditions provided clear mass spectra for every tested plant material. The identified compounds of the extracts belonged to various groups of metabolites: Phenolic acids, organic acids, flavonoids, flavonoid glucosides, tannins, sugars, vitamins, amino acids, and terpenoids. Certainly, the investigated hydroglycolic extracts were very rich sources of diverse polyphenols that had been characterized by marked biological potential, including antiradical properties. Both the peel and pulp of the investigated fruits contained valuable compounds; however, peel extracts were characterized by a greater variability of components. Nevertheless, all extracts—based on their compositional data—can be perceived as valuable sources of cosmetic ingredients that can exhibit beneficial properties for skin and hinder eventual skin care problems. The identification data are presented in the tables below, separately for every plant species, whereas the MS/MS spectra of the leading components are presented in the supplementary file in Table S1 (Supplementary Material).

Pulp and fruit extracts obtained from *A. cherimola* were rich in simple phenolic acids such as vanillic acid derivatives, but also in flavonoids (Table 2). Rutoside, catechin, epicatechin, procyanidin A and B, as well as kaempferol hexoside, were the major components of the tested extracts. Considering the part of the fruit, both flesh and peel contained a variety of flavonoids and phenolics. However, there were some minor compositional differences detected between the tested samples. Catechin, epicatechin, rutoside, and vanillic acid hexoside were identified only in the peel of the fruit, whereas procyanidins were traced in the pulp of the fruit. Similar classes of metabolites were detected in the leaves of the same plant species by Mannino et al. They used HPLC-DAD-ESI-MS/MS to identify 11 flavonoids in leaf extracts from *Annona* cultivars. Among the identified structures, six were flavonols (the glucosides and diglucosides of quercetin and kaempferol), three were flavones (the derivatives of luteolin and apigenin), and another two compounds belonged to the group of flavan-3-ols (catechin and epicatechin) [41]. In the investigated plant extracts, quercetin and kaempferol derivatives were detected next to the flavan-3-ols.

In the performed compositional studies, *Diospyros kaki* was found to have a different metabolite profile from the remaining fruits (Table 3). The persimmon extracts were rich in organic compounds and simple phenolics rather than in flavonoids. The HPLC-MS fingerprinting revealed the presence of quinic, citric, dehydroascorbic, and vanillic acids together with their glucosides, as well as glucogallin derivatives. Flavonoids catechin, epicatechin, and rutoside were detected in the peel of the fruit, whereas kaempferol glucoside and procyanidin A were present in the pulp. Similar constituents of kaki fruits were identified by Pu et al. [42], who listed gallic, ferulic, *p*-coumaric, vanillic acids, and catechin as the constituents of the investigated fruit extracts. Organic acids were detected in the fruits by Maulidiani et al. [43], whereas the juice was found to be rich in glucogallin, catechin, malic, vanillic, xylonic, and galacturonic acids [44].

The results of the chromatographic fingerprinting performed on the pulp and peel of *Cydonia oblonga* revealed the presence of flavonoids and phenolic acids. Among them, important and pharmacologically potent structures were identified, for example, rutoside, chlorogenic acid, kaempferol, luteolin, and quercetin glucosides, but also others (see Table 4). Certainly, the analyzed extracts from quince belong to flavonoid-containing ones. The samples are rich in the derivatives of quercetin, kaempferol, luteolin, and apigenin. In this case, a larger compositional variety between the peel and pulp extracts was observed than in other tested fruits. Peel was found to have a larger number of components in its extract. Among them, flavonoids such as vicenin-2, kaempferol rutinoside, or kaempferol galactoside were differentiating the samples. The described results are in accordance with previously published information. Formerly, the researchers described the presence of caffeoyl-quinic acids, quercetin, kaempferol, and its glucosides in the peeled and unpeeled quince jams [51]. In the studies on the aqueous extracts from the peel and pulp of *Cydonia oblonga* 5-*O*-caffeoylquinic acid constituted 57% and 29% of peel and pulp phenolic acids, respectively [52]. The former contained a wider diversity of secondary metabolites. Caffeoyl-quinic acid derivatives, rutoside, and quercetin glucosides were the major constituents of water peel extracts of the same plant species.

Fingerprinting of the last fruit, kumquat, that was also performed on the peel and pulp revealed the presence of various types of metabolites. HPLC-MS analysis provided high-resolution mass data for the recognition of flavonoids of different types, organic acids, and even a few terpenes in the extracts. The portfolio of flavonoid compounds present in *Fortunella margarita* is wide. In the plant extracts, we detected interesting flavonoids such as hesperidin, luteolinidin, vicenin, eriocitrin, rhoifolin, poncirin, fortunellin, and naringenin, together with sylforetin and acacetin glucosides (Table 5). The scientific literature shows many examples of compositional studies related to *Fortunella margarita.* According to other scientists, eriodictyol and apigenin were the major components of kumquat fruit, which—together with ascorbic acid, carotenoids, and dietary fiber—proved that the fruit is a good source of nutrients and non-nutrients [53]. The presence of the majority of the aforementioned flavonoids was confirmed by Kawaii et al. [54], except for quercetin and naringenin. Moreover, thirteen flavonoids were reported in the juice of kumquat that included different glucosides and diglucosides of acacetin, phloretin, narirutin, and naringin [55]. Also, spathulenol and carveol were detected previously in the plant when investigating the leaf essential oil by GC-MS methodology [56]. Here, these compounds were also visible in mass chromatograms recorded by a liquid chromatograph.

### 2.3. Antioxidant Potential of Pulp and Peel Extracts

Polyphenolic compounds are known mostly for their antioxidant potential. Effective neutralization of free radicals by polyphenols involves the donation of an electron or hydrogen atom, paring the unpaired electrons of free radicals, and reducing their reactivity. In the cosmetics industry, antioxidants are particularly important active ingredients due to their anti-aging and skin-protecting potential. Antioxidants are also used to prevent the oxidation of cosmetic constituents, leading to changes in the color, smell, and stability of cosmetic products [66].

**Figure 1:**
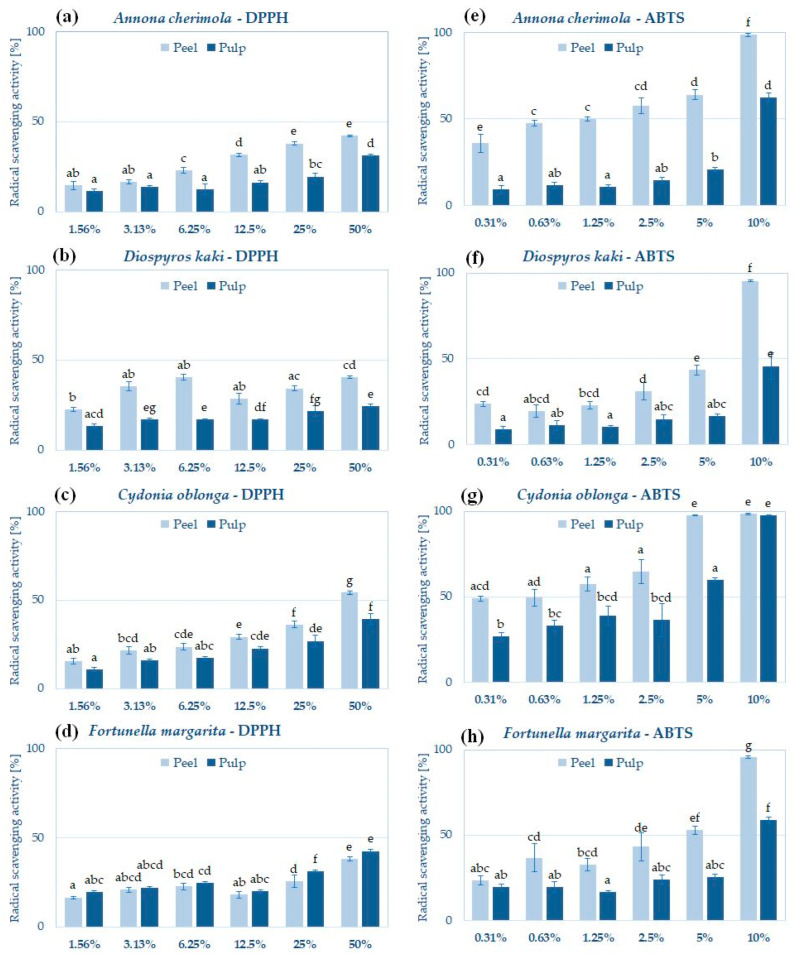
Antioxidant potential of peel and pulp extracts of *A. cherimola*, *D. kaki*, *C. oblonga* and *F. margarita* measured using DPPH (**a**–**d**) and ABTS (**e**–**h**) radical scavenging assays; histograms show mean ± SD; *n* = 3. The means not sharing the same letter are significantly different at *p* ≤ 0.05; Tukey’s post hoc test.

All analyzed extracts showed significant ABTS radical scavenging potential and lower activity in the DPPH scavenging assay (Figure 1). A similar pattern was observed by Zhang et al., who analyzed radical scavenging by *C. oblonga* fruit, leaf, and stem extracts [36]. According to Platzer et al., these differences may result from differential interactions of ABTS and DPPH radicals with natural compounds. For example, hydroxycinnamic acid and its derivatives achieve higher values than the hydroxybenzoic acids in the ABTS assay, whereas their activity in the DPPH assay is comparable. Some flavanones and dihydrochalcones are not reactive with the DPPH radical in contrast to the ABTS radical, leading to significant differences [67].

In almost all cases, peel extracts showed significantly higher radical scavenging potential than pulp extracts from the same fruit—with the exception of *F. margarita*, which pulp and peel extracts showed comparable activity in the reaction with DPPH (Figure 1d). The stronger antioxidant activity of hydroglycolic peel extracts agrees with the data obtained previously for alcoholic and hydroalcoholic extracts from the peels of cherimoya, persimmon, quince, and kumquat.

In both assays, the highest antioxidant activity was reported for *C. oblonga* peel extract (Figure 1c,g). This extract contained the highest levels of polyphenols among all the analyzed extracts, a high concentration of flavonoids, and significant amounts of proteins, which are more likely responsible for the observed radical scavenging effect. The high antioxidant activity of *C. oblonga* peel extract was previously reported by Hanan et al.—hydroethanolic extract from dried peel neutralized DPPH radical with an IC_50_ = 204.8 ± 2.24 μg/mL [38]. Silva et al. measured that the DPPH scavenging activity of methanolic quince peel extract (IC_50_ = 0.6 mg/mL) was higher than the activity of pulp and seed extract (IC_50_ of 1.7 and 2.0 mg/mL, respectively) [13]. In another study, Szychowski et al. analyzed the antioxidant activity of hydrophilic and lipophilic fractions of quince peel and pulp extracts by the ABTS radical scavenging assay and observed greater activity for the peel than for the pulp [39]. The antioxidant potential of *C. oblonga* peel extract has been correlated with the high content of polyphenolic compounds [14,60], which were also identified in the hydroglycolic extract of quince peel in this study (Table 4).

Albuquerque et al. showed that hydroethanolic extracts from the peels of Portuguese cherimoya cultivars display higher DPPH scavenging potential than the pulp and seed extracts from the same fruits. The compounds identified in these extracts, including lutein, β-cryptoxanthin, β-carotene, and vitamins C and E, were most likely responsible for the antioxidant effect [18]. Loizzo et al. showed using the DPPH and ABTS scavenging assays, beta-carotene bleaching test, Fe^2+^ chelation assay, and FRAP assay that the antioxidant properties of hydroethanolic peel extracts from Italian cherimoya cultivars were more significant than pulp extract [9].

Jeong et al. showed that the ABTS and DPPH radical scavenging activity of *D. kaki* peel extract strongly depends on the solvent used for extraction and the content of polyphenolics—in this study, the ethyl acetate extract showed the highest antioxidant activity [12]. In another study, persimmon peel extract in methanol and its fractions (especially the ethyl acetate fraction) were effective DPPH scavengers (IC_50_ = 23 µg/mL), whereas the aqueous extract showed rather low DPPH scavenging potential (IC_50_ > 400 µg/mL) [68]. The addition of persimmon peel extract was also effective in preventing lipid and protein oxidation in food products [69], suggesting that it might also be effective preservative for cosmetic formulations.

The antioxidant activity of the peel and pulp extracts of *F. margarita* has not been directly compared in the scientific literature to date. However, Sadek et al. investigated the DPPH scavenging activity of fractions obtained from the kumquat peel extracts using ethyl acetate, n-butanol, and dichlorometane—fractions obtained with ethyl acetate were the most active [15].

### 2.4. Skin Lightening Potential of Peel and Pulp Extracts—Inhibition of Tyrosinase

**Figure 2:**
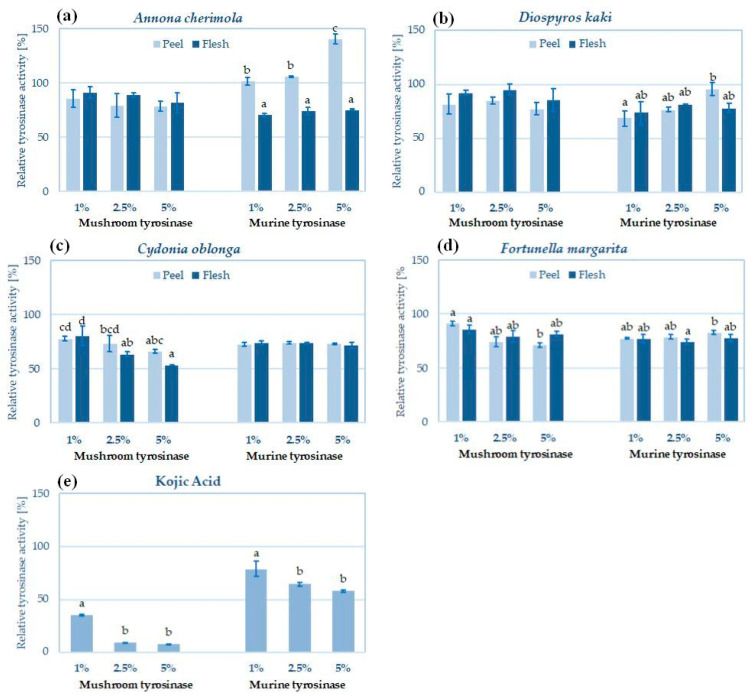
Inhibition of mushroom and murine tyrosinase activities by peel and pulp extracts of *A. cherimola* (**a**), *D. kaki* (**b**), *C. oblonga* (**c**) and *F. margarita* (**d**); kojic acid (**e**) was used as reference tyrosinase inhibitor; histograms show mean ± SD; *n =* 3. The means not sharing the same letter in each tyrosinase are significantly different at *p* ≤ 0.05; Tukey’s post hoc test.

Pigmentation disorders are one of the most common aesthetic problems, contributing to the growing popularity of skin-lightening cosmetics. Most cosmetic active ingredients that reduce hyperpigmentation are substances of natural origin, and their main target of action is tyrosinase (EC 1.14.18.1)—the cooper-containing oxidoreductase involved at the initial stages of melanogenesis. In the search for new skin-lightening substances, the commercially available mushroom tyrosinase inhibition test is most often used due to its high efficiency and low cost. However, mushroom and animal tyrosinases significantly differ in their structure and activity [70]. Therefore, the sensitivity of mushroom and animal enzymes to various inhibitors is sometimes also significantly different [71]. In this study, the tyrosinase inhibitory activity of hydroglycolic peel and pulp extracts was investigated using commercial mushroom tyrosinase and the lysate from melanoma cells as a source of murine tyrosinase (Figure 2). Detected mushroom and murine tyrosinase inhibitory activities result more likely from the synergistic action of flavonoids and phenolic acids present in the hydroglycolic pulp and peel extracts [72].

The most active in respect of tyrosinase inhibition was *C. oblonga* pulp extract, inhibiting the mushroom tyrosinase by 38–57% and the murine tyrosinase by 25–28% (Figure 2c). The inhibitory potential of *C. oblonga* peel extract was comparable (41–49% inhibition of mushroom tyrosinase and 26–27% of murine enzyme). The tyrosinase inhibitory potential of quince fruit extracts was previously reported, e.g., by Khiljee et al., who detected 90% mushroom tyrosinase inhibition by 1 mg/mL of *C. oblonga* whole fruit extract [27]. Fruit, stem, and leaf extracts from *C. oblonga* were also described as effective mushroom tyrosinase inhibitors by Zhang et al., with tyrosinase inhibitory potentials of 56.64, 72.11, and 68.32 mg kojic acid equivalent (KAE)/g, respectively) [36]. Previous studies of hydroalcoholic extracts described flavonoids, anthocyanins, and lignans as potent tyrosinase inhibitors of quince extracts [36,73]. In this study, the influence of quince extract on animal tyrosinase was investigated for the first time.

Interesting results were obtained for *A. cherimola* extracts (Figure 2a). The pulp extract inhibited both mushroom and murine tyrosinase by 25–34% (no significant differences between tyrosinases). The peel extract showed similar mushroom tyrosinase inhibitory levels (38–40%) but increased the activity of murine tyrosinase by up to 40% at the highest tested concentration (5%, *v/v*). Several plant extracts and natural compounds have been described in the literature to date to increase the activity of tyrosinase and enhance melanin biosynthesis. These ingredients are potential treatment options for hypopigmentation disorders, such as vitiligo [74]. In previous studies, *A. cherimola* peel extracts were tested only using the mushroom enzyme—80% ethanol extract from the peel weakly inhibited mushroom tyrosinase with an IC_50_ = 120 mg/mL, but the compounds responsible for this activity were not identified [22]. Due to the lack of studies describing the influence of *A. cherimola* extracts on melanin synthesis and tyrosinase activity in animal models, further investigations should be performed to elucidate the opposite action of *A. cherimoya* pulp and peel extracts on the activity of murine tyrosinase.

Comparable tyrosinase inhibitory potential was detected in the peel and pulp extracts from *D. kaki* (Figure 2b). Both extracts were more active towards mushroom tyrosinase (34–37% and 25–30% inhibition for peel and pulp, respectively) and less active toward murine enzyme (max. 31% and 26% inhibition for mushroom and murine tyrosinases, respectively). In another study, Fukai et al. identified 2-methoxy-4-vinylphenol as an effective mushroom tyrosinase inhibitor of persimmon peel (IC_50_ with L-tyrosine = 0.5 mM and with L-DOPA = 0.7 mM), more active than refence tyrosinase inhibitor arbutin (IC_50_ with L-tyrosine = 0.7 mM, with L-DOPA = 2.9 mM) [68]. The skin-lightening potential of persimmon peel extracts was previously investigated by Ohguchi et al. for their inhibitory activity on melanin biosynthesis in mouse B16 melanoma cells. The activity-guided purification of the extract resulted in the isolation of two flavonoid glycosides, isoquercitrin (quercetin-3-*O*-glucoside) and hyperin (quercetin-3-*O*-galactoside), that strongly inhibited the production of melanin (IC_50_ values of 21.7 and 18.2 µM, respectively), but they did not influence the activity of intracellular tyrosinase. The inhibitory effect was found to be mediated by suppressing tyrosinase expression [75].

In respect of *F. margarita* pulp extract (Figure 2d), weak tyrosinase inhibitory activity was detected (29–32% and 23–26% inhibition for mushroom and murine tyrosinase, respectively). The peel extract was slightly more active on mushroom tyrosinase (31–40% inhibition) and slightly less on murine enzyme (18–23% inhibition). The impact of *F. margarita* extracts on tyrosinase activity or melanin biosynthesis has not been described in the scientific literature to date; however, some active compounds identified in the hydroglycolic pulp and peel extracts (e.g., hesperidin, naringenin, quercetin) are known as efficient mushroom tyrosinase inhibitors [76,77].

### 2.5. Sun Protecting Potential of Flesh and Peel Extracts—SPF In Vitro

Ultraviolet radiation (UVR) has some positive impacts on human health (e.g., mood improvement and biosynthesis of vitamin D_3_), but it is also a cause of several harmful effects, including hyperpigmentation, sunburn, photoaging, and an increased risk of skin cancer occurrence [78]. The most effective method of preventing the harmful effects of UVR is the use of sunscreens, commonly incorporated into the formulations of modern cosmetic products. The efficacy of a sun-protecting cosmetic is usually expressed as the sun protection factor (SPF), defined as the UVR energy required for inducing a minimal erythema dose (MED) on protected skin, divided by the UVR energy required for inducing MED on unprotected skin. The application of synthetic sunscreens raises some concerns, regarding their safety, natural sunscreens are currently extensively investigated as safer alternatives [79]. For that reason, the pulp and peel fruit extracts were also analyzed for their sun-protecting potential in vitro. As shown in Table 6, the highest SPF values were calculated for kumquat extracts, ranging from 6.41 ± 0.77 to 7.48 ± 0.40 for pulp and peel extracts, respectively, at the highest tested concentration (5%, *v/v*). *F. margarita* peel and pulp extracts might be considered effective photoprotective cosmetic ingredients due to the presence of phytocompounds with known photoprotective activity—carotenoids and flavonoids [80].

### 2.6. The Influence of Peel and Pulp Extracts on the Viability of Skin Fibroblasts and Keratinocytes—In Vitro Cytotoxicity

**Figure 3:**
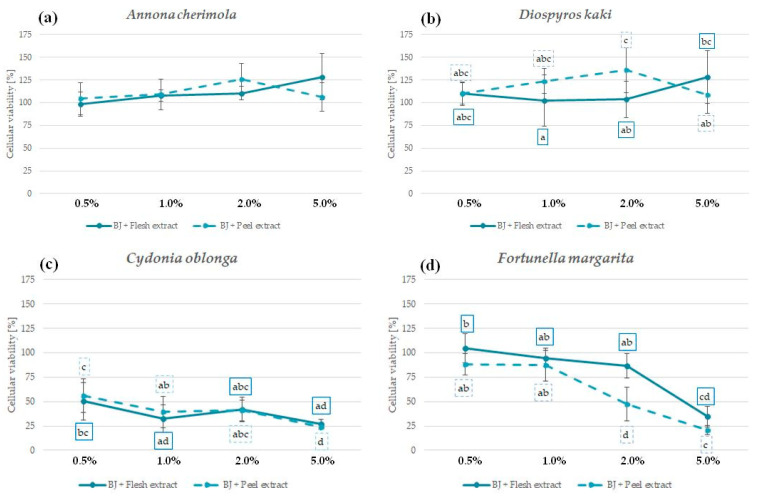
Cytotoxicity of *A. cherimola* (**a**), *D. kaki* (**b**), *C. oblonga* (**c**), and *F. margarita* (**d**) pulp and peel extracts against human BJ fibroblasts following 48 h exposure; graphs show mean ± SD; *n =* 3. The means not sharing the same letter are significantly different at *p* ≤ 0.05; Tukey’s post hoc test.

**Figure 4:**
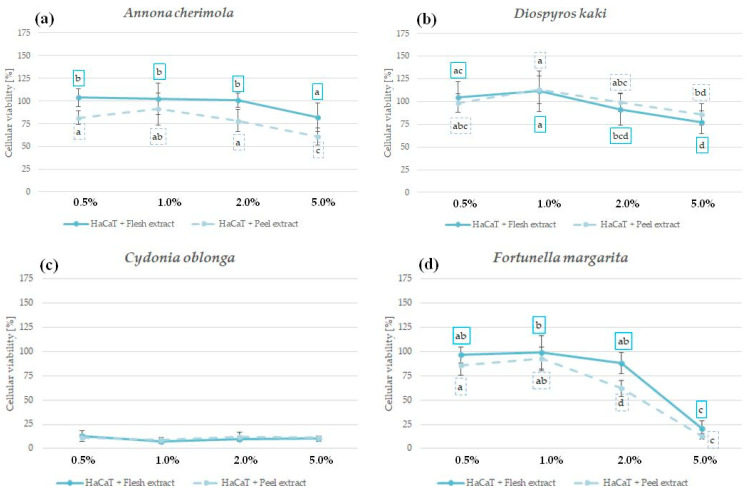
Cytotoxicity of *A. cherimola* (**a**), *D. kaki* (**b**), *C. oblonga* (**c**), and *F. margarita* (**d**) pulp and peel extracts against human keratinocytes HaCaT following 48 h exposure; graphs show mean ± SD; *n =* 3. The means not sharing the same letter are significantly different at *p* ≤ 0.05; Tukey’s post hoc test.

In order to confirm the safety and lack of irritation potential, the cytotoxic effect of analyzed peel and pulp extracts was investigated in vitro using human fibroblasts BJ (Figure 3) and human immortalized keratinocytes HaCaT (Figure 4) [81]. The influence of peel and pulp extract of the same fruit on both cell lines was comparable; however, HaCaT keratinocytes were slightly more sensitive to the extract treatment.

No significant reduction in fibroblast viability was observed for *A. cherimola* and *D. kaki* extracts at the tested concentration range. Interestingly, *D. kaki* peel extract significantly increased the number of viable fibroblasts at 1% and 2%, suggesting its regenerative potential (Figure 3a,b). Extracts from the peels and pulps of both species were also relatively safe for HaCaT keratinocytes—cellular viability at all tested concentrations was >50% (Figure 4a,b). Based on the presented results and the lack of other data showing the cytotoxic potential of *A. cherimola* and *D. kaki* extracts, it could be concluded that hydroglycolic pulp and peel extracts from these fruit species are safe to use for topical applications.

*C. oblonga* extracts were highly cytotoxic for both cell lines, reducing the viability of fibroblasts below 50% and the viability of keratinocytes below 25% at all analyzed concentrations (Figure 3c and Figure 4c). In previous study, *C. oblonga* peel extract enriched in polyphenols was cytotoxic also for NIH/3T3 murine fibroblasts at 10 and 20 mg/mL [82]. The cytotoxic activity of *C. oblonga* extracts against fibroblasts and keratinocytes suggests that topical application of these extracts might cause skin irritation. However, the emulgel containing 4% of total fruit quince extracts was shown to be nonirritant in a 48 h patch test, and the volunteers using the cosmetics for up to 12 weeks did not report any adverse effects [27].

*F. margarita* extracts were not significantly cytotoxic for both cell lines up to a concentration of 2%. At 5%, the viability of fibroblasts and keratinocytes was reduced to ca. 25% (Figure 3d and Figure 4d). For both tested cell lines, the *F. margarita* peel extract was more cytotoxic than the pulp extract, raising possible concerns regarding its safety following topical application.

### 2.7. In Vitro Cytotoxicity of Peel and Pulp Extracts on Human Melanoma Cells

**Figure 5:**
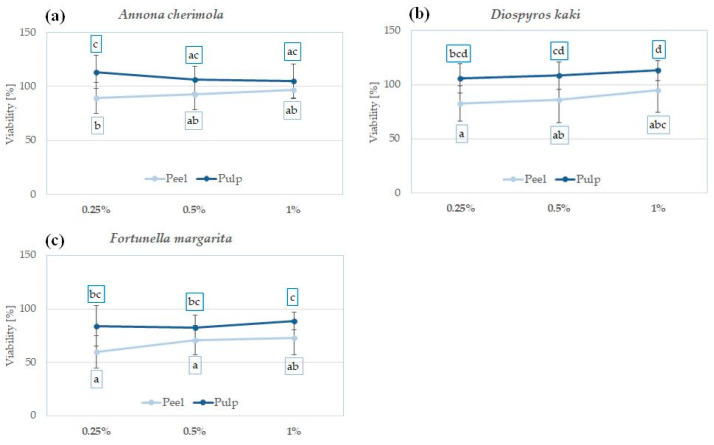
Cytotoxicity of *A. cherimola* (**a**), *D. kaki* (**b**), and *F. margarita* (**c**) peel and pulp extracts against human melanoma cell line A375; graphs show mean ± SD; *n =* 3. The means not sharing the same letter are significantly different at *p* ≤ 0.05; Tukey’s post hoc test.

In order to explore their chemopreventive potential, *A. cherimola*, *D*. *kaki*, and *F. margarita* peel and pulp extracts were analyzed for their cytotoxicity towards the human malignant melanoma cell line A375 (Figure 5), using concentrations that were nontoxic for non-cancerous skin cells in the same experimental conditions (Figure 3 and Figure 4). *C. oblonga* extracts were not included in this study due to their previously described toxicity. For all the analyzed fruit species, the pulp extracts showed no significant reduction in the viability of A375 cancer cells. All the analyzed peel extracts showed significantly higher cytotoxic effects in at least one of the tested concentrations. *F. margarita* peel extracts were the most cytotoxic, reducing the viability of A375 cells by ca. 25% at 1% (Figure 5c).

The influence of peel, pulp, or whole fruit extracts of cherimoya, persimmon, quince, and kumquat on the viability of melanoma cells in vitro was not previously described in the scientific literature. In respect of *A. cherimola*, one study showed significant cytotoxicity of the leaf extract towards human malignant melanoma cell lines A2058 and Sk-Mel-28 and lower cytotoxic activity in non-cancerous 3T3-L1 fibroblasts. The extract also decreased melanoma cell migration, triggered apoptosis, and disrupted the organization of the cytoskeleton [83].

## 3. Materials and Methods

### 3.1. Chemicals and Reagents

Glycerine (>99.8%), Folin–Ciocalteau reagent, and K_2_S_2_O_8_ were obtained from Chempur (Piekary Śląskie, Poland). Ethanol (>99.8%) was obtained from Honeywell (Charlotte, NC, USA). Tyrosinase from *Agaricus bisporus*, 3,4-dihydroxy-l-phenylalanine (L-DOPA), 2,2-diphenyl-1-picrylhydrazyl (DPPH), 2,2′-azino-bis(3-ethylbenzothiazoline-6-sulfonic acid (ABTS), kojic acid (≥98.5% purity), quercetin (>95% purity), gallic acid (>97.5%), Dulbecco’s phosphate buffered saline (DPBS), Dulbecco’s modified Eagle’s medium (DMEM), bovine serum albumin (BSA), neutral red solution in DPBS (3.3 g/L) were purchased from Sigma Aldrich (Merck, Darmstadt, Germany). Fetal bovine serum (FBS) was obtained from Pan-Biotech (Aidenbach, Germany). The solvents used for phytochemical studies by HPLC-MS (water, acetonitrile, and formic acid) were purchased from Merck (Darmstadt, Germany).

### 3.2. Plant Material and Extract Preparation

Ripe fruits of *Annona cherimola*, *Diospyros kaki*, *Cydonia oblonga*, and *Fortunella margarita* were purchased from a local fruit supplier. The fruits were washed, peeled, and dried at a temperature not exceeding 45 °C. Voucher specimens of dried plant materials are kept at the Department of Cosmetology, University of Information Technology and Management in Rzeszow, Poland. Two grams of dried, ground plant material (mesocarp—pulp or exocarp—peel) was mixed with 100 mL of water:PG (1:1, *v/v*) mixture and subjected to ultrasound-assisted extraction for 1 h at room temperature using an ultrasonic bath (Sonic-3, Polsonic, Warsaw, Poland). The extracts were filtered through filter paper and a 0.45 µm syringe filter and stored at −20 °C until analysis.

### 3.3. The Content of Total Polyphenols, Flavonoids and Proteins

The content of total polyphenols and flavonoids in the obtained extracts was determined according to the methods described by Fukumoto and Mazza [84] and Matejić et al. [85], respectively. For polyphenol determination, 150 μL of 10% (*v/v*) extracts was mixed with 750 μL of Folin–Ciocalteu reagent (1:10, *v/v*, in distilled water). Following a 5 min incubation at room temperature, the samples were mixed with 600 μL 7.5% (*m/v*) Na_2_CO_3_ and incubated for another 60 min. The absorbance was measured at λ = 740 nm using a DR 600 spectrophotometer (Hach Lange, Wrocław, Poland). The calibration curve was established using gallic acid (0–100 mg/mL), and the concentration of total polyphenols was displayed as µg gallic acid equivalents (GAE) per mL of the extract. For measurement of flavonoid content, 150 μL 10% (*v/v*) extracts was mixed with a 650 μL reaction mixture containing 61.5 mL 80% (*v/v*) ethanol, 1.5 mL 10% aluminum nitrate nonahydrate Al(NO_3_)_3_·9H_2_O, and 1.5 mL 1 M potassium acetate CH_3_COOK. The absorbance of the samples was measured at λ = 415 nm following a 40 min incubation at room temperature in darkness. The calibration curve was established using quercetin (0–100 mg/mL), and the content of flavonoids was expressed as µg quercetin equivalents (QE) per mL of the extract. Protein concentration in the extracts was measured by Bradford protein assay [86] and displayed as mg albumin equivalents (AE) in 1 mL of the extract.

### 3.4. HPLC-ESI-QTOF-MS/MS Fingerprinting of the Obtained Extracts

The identification of single components of the analyzed extracts was performed using an HPLC-MS chromatograph produced by Agilent Technologies (Santa Clara, CA, USA). The chromatograph (1200 Series) built from the following segments: A binary pump, a degasser, an autosampler, a column thermostat, and a UV detector was coupled with a Q-TOF-MS/MS detector with electrospray ionization that was operated in both positive and negative ionization mode (Agilent Technologies, G6530B Series). The identification of plant metabolites was performed using the Mass Hunter program (version B.10.00) that was delivered with the instrumentation and used the recorded high-accuracy mass measurement data, the inbuilt molecular formula calculators (mass measurement error tolerance of 15 ppm), but also relied on the scientific literature and mass spectrometric databases.

The fractionation of the analyzed extracts was performed on the ReproSil-Pur 120 C18-AQ column (Shimadzu, Kyoto, Japan) with a 5 um pore diameter and dimensions of 150 mm × 4.6 mm, in the same gradient of acetonitrile with 0.1% formic acid (solvent B) in 0.1% aqueous solution of formic acid (solvent A). The following program was applied: 0 min: 1% of B in A, 2 min: 1% of B in A, 10 min: 10% of B in A, 18 min: 40% of B in A, 22–27 min: 95% of B in A, 27.1–35 min: 1% of B in A. The flow rate was set at 0.2 mL, the temperature at 20 °C, and the post-time at 5 min. A mass spectrometer was operated at the following settings: *m/z* range: 40–2500 Da, number of precursors per cycle: 2, gas and sheath gas temperatures of 275 and 325 °C, respectively, the gas flows of 12 L/min, nebulizer pressure of 35 psig, the capillary voltage of 3000 V, nozzle voltage of 1000 V, the fragmentor voltage of 110 V, the skimmer voltage of 65 V, and the collision energy of 10 V. The selected peak areas of the leading components of the extracts were taken directly from the program and constitute relative intensity information. The extracts were injected in triplicate in both negative and positive ionization modes.

### 3.5. Antioxidant Activity—DPPH and ABTS Scavenging Assays

The antioxidant capacity of flesh and peel extracts was compared using the 2,2-diphenyl-1-picrylhydrazyl (DPPH) and 2,2′-azino-bis(3-ethylbenzothiazoline-6-sulfonic acid (ABTS), scavenging assays, according to the protocols described by Matejić et al. [85], with further modifications [87]. The extracts were analyzed in a concentration range of 0–50% (*v/v*), and the percentage of radical scavenging for each concentration was calculated from the formula below:

% of ABTS/DPPH scavenging = [1 − (Abs(S)/Abs(C))] × 100

where Abs(S)—the corrected absorbance of the extract, Abs(C)—the corrected absorbance of the control sample.

### 3.6. Tyrosinase Inhibitory Assay

The tyrosinase inhibitory potential of flesh and peel extracts was compared using commercially available mushroom tyrosinase and murine tyrosinase present in the lysate of murine melanoma cell B16F10 (ATCC CRL-6475; LGC Standards, Łomianki, Poland), prepared as previously described [71]. For the mushroom tyrosinase inhibitory assay, 20 μL of diluted extracts (5%, 2.5%, and 1%) were mixed with 120 μL phosphate buffer (100 mM, pH = 6.8) and 20 μL of mushroom tyrosinase working solution (500 U/mL). Following a 10 min pre-incubation at room temperature, 40 μL L-DOPA (4 mM) was added, and the samples were kept for another 20 min at RT. The activity of murine tyrosinase was assessed by mixing 20 µL of diluted extract (5%, 2.5%, and 1%), 40 µL 4 mM L-DOPA, and 100 mM phosphate buffer pH 6.8 with a volume of B16F10 lysate containing 20 µg protein. The reaction was carried out for 4 h incubation at 37 °C. The control sample (100% tyrosinase activity) for both assays contained an appropriate volume of the solvent instead of the extract. Kojic acid in equal concentrations was used as a known tyrosinase inhibitor control. In both assays, the dopachrome formation was measured spectrophotometrically at λ = 450 nm using a FilterMax F5 microplate reader(Molecular Devices, San Jose, CA, USA). The obtained values were corrected by the absorbance values of the extracts without mushroom or murine tyrosinase and L-DOPA. Relative murine or mushroom tyrosinase activity (in %) was measured in comparison with the appropriate control sample.

### 3.7. Determination of the Sun Protection Factor (SPF)

The sun protection factor (SPF) of flesh and peel extracts at 5%, 2.5%, and 1% (*v/v*) was estimated based on the absorbance values (λ = 290–320 nm) measured using the DR600 UV-Vis spectrophotometer (Hach Lange, Wrocław, Poland), the EE × I values determined by Sayre [88], and the Mansur Equation [89]:

$$\mathrm{SPF}=\mathrm{CF}\;\times \;\sum_{290}^{320}EEx\left(\lambda \right)xIxAbs(\lambda )$$

where EE (λ)—erythemal effect spectrum; I (λ)—solar intensity spectrum; Abs (λ)—absorbance of the sample; CF—correction factor (=10).

### 3.8. In Vitro Cytotoxicity

The cytotoxic effect of flesh and peel extracts was analyzed on human A375 malignant melanoma, BJ fibroblasts (LGC Standards, Łomianki, Poland), and HaCaT keratinocytes (CLS Cell Lines Service GmbH, Eppelheim, Germany) using the neutral red uptake test [90]. BJ cells were grown in EMEM, A375 and HaCaT cells were maintained in DMEM, supplemented with 10% (*v/v*) FBS at 37 °C in a humidified atmosphere with 5% CO_2_. For the experiments, the cells were plated onto 96-well plates (1 × 10^4^ cells/well) and, following overnight incubation, treated for 48 h with 5%, 2%, 1%, and 0.5% of the extracts. Control cells were grown in the presence of equal volumes of the solvent (H_2_O:PG, 1:1, *v/v* at 5%, 2%, 1% and 0.5%). The neutral red solution (33 µg/mL) diluted in appropriate culture media was incubated with the cells for 2 h. The cells were then lysed in an acidified ethanol solution (50% *v/v* ethanol, 1% *v/v* acetic acid, and 49% H_2_O). The absorbance of the released neutral red was measured at λ = 540 nm using a FilterMax F5 microplate reader (Molecular Devices, San Jose, CA, USA). The mean absorbance value of the control cells for each extract concentration was set at 100% viability and used to calculate the percentage of viable cells following extract treatment.

### 3.9. Statistical Analysis

All analyses were performed in three replicates. The final results are presented as the mean ± standard deviation (S.D.). Differences between the groups were analyzed with a student’s *t*-test or one-way ANOVA or two-way ANOVA followed by a Tukey’s post hoc test. Results were considered statistically significant when *p* < 0.05. Statistical analyses were performed using Statistica software v13.3 (StatSoft, Krakow, Poland).

## 4. Conclusions and Future Perspectives

Hydroglycolic extracts from the peels might replace the extracts obtained from the edible parts of *A. cherimoya*, *D. kaki*, *C. oblonga*, and *F. margarita* in cosmetic formulations, as they showed better, or at least comparable, cosmetic-related properties to the pulp extracts from the same fruits. *A. cherimola*, *D. kaki*, and *C. obonga* peel extracts contained several times more polyphenols than pulp extracts, and *A. cherimola*, *C. oblonga*, and *F. margarita* peel extracts contained more flavonoids. The phytochemical composition of all peel extracts was richer, containing some unique polyphenolic compounds that were not extracted from the fruit pulp. All peel extracts showed significantly higher antiradical potential than pulp extracts, suggesting their potent application in anti-aging and skin-protecting cosmetic products. *C. oblonga* extracts were the most potent skin-lightening ingredients due to the significant inhibitory activity of murine tyrosinase, comparable with 1% kojic acid. *F. margarita* peel extract might be used as a skin-lightening ingredient and natural sunscreen due to significant tyrosinase inhibition and in vitro SPF values. *A. cherimola* pulp and peel extracts might be applied in topical preparations for the treatment of various types of pigmentation disorders, as they inhibit (pulp extract) or activate (peel extract) the tyrosinase. Interestingly, the cytotoxicity of peel and pulp extracts was comparable toward non-cancerous skin cells in vitro (fibroblasts and keratinocytes), whereas peel extracts were more cytotoxic for melanoma cells than pulp extracts at the same concentration range. The data from in vitro cytotoxicity studies indicated that further studies are needed to elucidate the safety and irritation potential of *C. oblonga* peel and pulp extracts, e.g., using 3D epidermal models or patch tests.

Application of fruit peel extracts in cosmetic products also requires careful preparation of the plant material in order to avoid extract contamination with pesticides. This might be obtained by washing the peels in water containing 2% or 6% acetic acid, as acidified solutions are able to chelate most of the commonly used pesticides and effectively remove their traces from the plant material [91,92].

## Figures and Tables

**Table 1:** The total polyphenol content (TPC), the total flavonoids content (TFC) and the protein content in hydroglycolic extracts from pulp and peel of *A. cherimola*, *D. kaki*, *C. oblonga*, and *F. margarita*; mean ± SD, *n* = 3. The symbol *** indicates that the peel differs from the pulp in the content of the tested group of compounds for a given species; *p* ≤ 0.001; Student’s *t*-test (GAE—gallic acid equivalents; QE—quercetin equivalents; AE—albumin equivalents).

	*Annona cherimola*		*Diospyros kaki*		*Cydonia oblonga*		*Fortunella margarita*	
	Pulp	Peel	Pulp	Peel	Pulp	Peel	Pulp	Peel
TPC [µg GAE/mL]	357.06 *** ± 14.49	1596.06 ± 31.68	224.14 *** ± 5.36	778.31 ± 16.55	1188.28 *** ± 23.35	1814.94 ± 54.19	1299.39 ± 79.59	1341.06 ± 23.59
TFC [µg QE/mL]	3.38 *** ± 0.88	104.63 ± 3.31	nd	nd	nd	222.75 ± 6.50	81.50 *** ± 1.77	301.92 ± 48.16
Protein [µg AE/mL]	nd	nd	nd	4.45 ± 0.25	27.39 ± 3.57	28.65 ± 0.56	nd	nd

**Table 2:** Compounds identified in pulp and peel extracts of *Annona cherimola* together with the mass chromatograms obtained for the pulp (left) and peel (right) extracts in both negative (above) and positive (below) ionization modes (Mexp—experimental mass, Mcalc—theoretical mass, error—error of measurement, DBE—double bond equivalent number, + present, − absent).

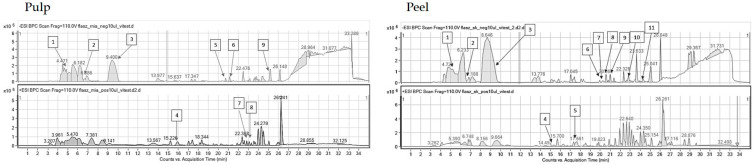												
No.	RT [min]	Structure	Mexp.	Mcalc	Ions	Name	Error [ppm]	DBE	Compound Class	Ref.	*Annona cherimola*	
											Pulp	Peel
1	4.27	C_6_H_12_O_6_	179.055	179.0561	[M-H]−	Fructose	7.17	1	Organic compound	[10,45]	+	+
2	4.69	C_7_H_12_O_6_	191.0542	191.0561	[M-H]−	Quinic acid	9.95	2	Organic compound	[10,45]	+	+
3	5.47	C_5_H_9_NO_2_	116.0711	116.0706	[M-H]+	Proline	0.04	2	Amino acids	[10,45]	+	+
4	6.01	C_6_H_8_O_7_	191.0189	191.0197	[M-H]−	Citric acid	4.30	3	Organic compound	[10,45]	+	+
5	8.69	C_6_H_6_O_6_	173.0096	173.0092	[M-H]−	Dehydroascorbic acid	9.51	3	Organic compound	[10,45]	+	+
6	19.3	C_15_H_14_O_6_	289.0695	289.0718	[M-H]−	Catechin	7.8	9	Flavonoids	[10,45,46]	−	+
7	20.42	C_16_H_22_O_10_	373.1116	373.114	[M-H]−	3-beta-glucopyranosyloxy-2-hydroxy-1-(4-hydroxy-3-methophenyl)-propan-1-one	6.47	6	Phenolic compound	[10,45]	+	+
8	20.11	C_14_H_18_O_9_	329.0896	329.0878	[M-H]−	Vanilic acid hexoside	−5.44	6	Glucosides	[10,45]	−	+
9	20.35	C_15_H_14_O_6_	289.0691	289.0718	[M-H]−	Epicatechin	9.18	9	Flavonoids	[10,45,46]	−	+
10	20.52	C_14_H_20_O_8_	315.1089	315.1085	[M-H]−	Hydroxytyrosol 4-*O*-glucoside	−1.14	5	Phenolic compound	[10,45]	+	+
11	20.82	C_30_H_24_O_12_	577.1392	577.1311	[M-H]−	Procyanidin type B	−7.01	18	Tannins	[10,45]	+	+
12	22.93	C_32_H_38_O_20_	741.1862	741.1884	[M-H]−	Calabricoside	2.92	14	Phenolic compound	[10,45]	+	+
14	23.84	C_27_H_30_O_16_	609.1454	609.1461	[M-H]−	Rutoside	1.16	13	Phenolic compound	[45]	−	+
15	25.09	C_30_H_24_O_12_	575.1206	575.1195	[M-H]−	Procyanidin type A	−1.91	19	Tannins	[10,45]	+	−
16	25.19	C_21_H_19_O_11_	447.0922	447.0933	[M-H]−	Kaempferol hexoside I	2.42	12	Phenolic compound	[10,45]	+	−

**Table 3:** Compounds identified in pulp and peel extracts of *Diospyros kaki* together with the mass chromatograms obtained for the pulp (left) and peel (right) extracts in both negative (above) and positive (below) ionization modes (Mexp—experimental mass, Mcalc—theoretical mass, error—error of measurement, DBE—double bond equivalent number, + present, − absent).

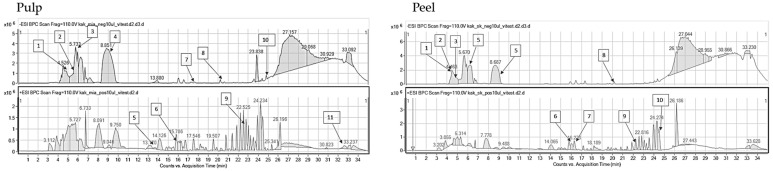												
No.	RT [min]	Structure	Mexp.	Mcalc	Ions	Name	Error [ppm]	DBE	Compound Class	Ref.	*Diospyros kaki*	
											Pulp	Peel
1	4.51	C_6_H_12_O_7_	195.0491	195.0510	[M-H]−	Glucuronic acid	9.83	1	Organic acids	[43]	+	+
2	4.53	C_6_H_10_O_7_	193.0360	193.0354	[M-H]−	Hexuronic acid	−3.21	2	Organic acids	[47]	+	+
3	4.56	C_5_H_10_O_6_	165.0420	165.0405	[M-H]−	Xylonic acid	−9.26	1	organic acids	[43]	+	+
4	5.77	C_4_H_6_O_5_	133.0157	133.0142	[M-H]−	Malic acid	−5.62	2	Organic acids	[43]	+	+
5	8.85	C_6_H_8_O_7_	191.0198	191.0197	[M-H]−	Citric acid	−0.38	3	Organic acids	[43]	+	+
6	14.07	C_4_H_6_O_4_	117.0183	117.0193	[M-H]−	*p*-coumaric acid	8.75	2	Organic acid	[48]	+	-
7	22.8	C_15_H_14_O_6_	289.0684	289.0718	[M-H]−	Epicatechin	11.59	9	Flavonoids	[43]	+	+
8	16.33	C_13_H_16_O_10_	331.0702	331.0671	[M-H]−	Glucogallin isomer 1	−9.42	6	Phenolic compound	[43]	+	+
9	18.05	C_7_H_6_O_5_	169.0155	169.0142	[M-H]−	Gallic acid	−7.37	5	Phenolic acid	[48]	+	+
10	20.22	C_13_H_16_O_10_	331.0702	331.0671	[M-H]−	Glucogallin isomer 2	−9.42	6	Phenolic compound	[43]	+	+
11	20.36	C_8_H_8_O_4_	167.0368	167.035	[M-H]−	Vanillic acid	−10.82	5	Phenolic acids	[48,49]	−	+
12	22.66	C_10_H_10_O_4_	195.0632	195.0652	[M-H]+	Ferulic acid	10.23	6	Phenolic acids	[48,49]	+	+
13	24.84	C_21_H_24_O_10_	435.1319	435.1297	[M-H]−	Phloridzin	−5.11	10	Phenolic compound	[42]	+	−
14	24.93	C_15_H_10_O_4_	287.0549	287.055	[M-H]+	Kaempferol	0.40	11	Flavonoids	[50]	−	+
15	25.0	C_8_H_14_O_4_	173.0828	173.0819	[M-H]−	Propylglutaric acid	−4.98	2	Organic acids	[43]	+	+
16	33.14	C_8_H_6_O_4_	165.022	165.0193	[M-H]+	*O*-phtalic acid	−16.07	6	Organic compound	[49]	+	−

**Table 4:** Compounds identified in pulp and peel extracts of *Cydonia oblonga* together with the mass chromatograms obtained for the pulp (left) and peel (right) extracts in both negative (above) and positive (below) ionization modes (Mexp—experimental mass, Mcalc—theoretical mass, error—error of measurement, DBE—double bond equivalent number, + present, − absent).

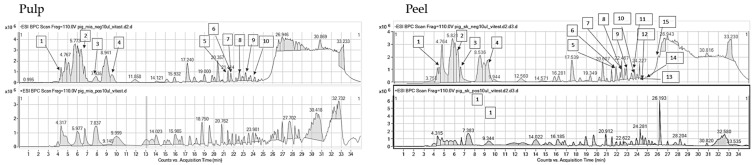												
No.	RT [min]	Structure	Mexp.	Mcalc	Ions	Name	Error [ppm]	DBE	Compound Class	Ref.	*Cydonia oblonga*	
											Pulp	Peel
1	4.77	C_7_H_12_O_6_	191.0565	191.0561	[M-H]−	Quinic acid	−2.02	2	Organic acids	[39]	+	+
2	6.36	C_4_H_6_O_5_	133.0149	133.0142	[M-H]−	Malic acid	−4.87	2	Organic acids	[39,57]	+	+
3	6.64	C_12_H_18_O_11_	337.0778	337.0776	[M-H]−	Ascorbyl glucoside	−0.49	4	Glucosides	-	+	+
4	9.49	C_6_H_8_O_7_	191.0208	191.0197	[M-H]−	Citric acid	−5.59	3	Organic acids	[39]	+	+
5	21.31	C_16_H_18_O_9_	353.0869	353.0878	[M-H]−	Chlorogenic bxsacid	2.56	8	Phenolic acids	[52,57]	+	+
6	21.45	C_18_H_16_O_9_	375.0706	375.0722	[M-H]−	Limocitrol	4.14	11	Flavonoids	[58]	+	+
7	22.43	C_16_H_18_O_9_	353.0879	353.0878	[M-H]−	Neochlorogenic acid	−0.27	8	Phenolic acids	[52,57]	+	+
8	23.13	C_16_H_18_O_9_	353.0882	353.0878	[M-H]−	(*Z*)-chlorogenic acid	−1.11	8	Phenolic acids	[52,57]	−	+
9	23.68	C_27_H_30_O_16_	609.1462	609.1461	[M-H]−	Rutoside	−0.15	13	Flavonoids	[52]	+	−
10	23.8	C_19_H_30_O_18_	449.1707	449.1664	[M-H]−	Luteolin glucoside	−9.44	5	Flavonoids	[36]	+	+
11	24.29	C_27_H_30_O_15_	593.1512	593.1509	[M-H]−	Vicenin-2	0.65	13	Flavonoids	[59]	−	+
12	26.29	C_30_H_26_O_13_	593.1301	593.1301	[M-H]−	Kaempferol 3-rutinoside	−0.06	18	Flavonoids	[60]	−	+
13	24.34	C_21_H_20_O_12_	463.0919	463.0882	[M-H]−	Quercitin-3-galactoside	−7.97	12	Flavonoids	[60]	+	+
14	24.94	C_21_H_20_O_12_	447.0933	447.0933	[M-H]−	Kaempferol-3-*O*-galactoside	−0.48	12	Flavonoids	[60]	−	+

**Table 5:** Compounds identified in pulp and peel extracts of *Fortunella margarita* together with the mass chromatograms obtained for the pulp (left) and peel (right) extracts in both negative (above) and positive (below) ionization modes (Mexp—experimental mass, Mcalc—theoretical mass, error—error of measurement, DBE—double bond equivalent number, + present, − absent).

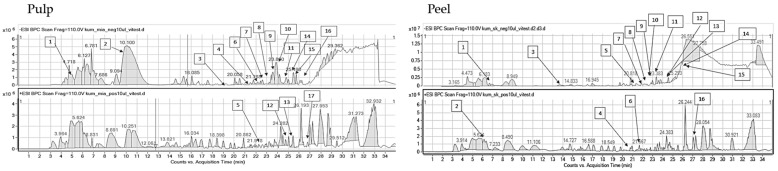												
No.	RT [min]	Structure	Mexp.	Mcalc	Ions	Name	Error [ppm]	DBE	Compound Class	Ref.	*Fortunella margarita*	
											Pulp	Peel
1	24.72	C_28_H_34_O_15_	609.1831	609.1884	[M-H]−	Hesperidin	−0.99	12	Flavonoids	[29,61]	+	−
2	6.58	C_15_H_10_O_7_	301.0353	301.0354	[M-H]−	Quercetin	0.25	11	Flavonoids	[61]	−	+
3	6.73	C_15_H_11_O_5+_	272.0684	272.0684	[M-H]−	Luteolinidin	2.28	9.5	Flavonoids	[62]	−	+
4	10.05	C_6_H_8_O_7_	191.0172	191.0197	[M-H]−	Citric acid	13.16	3	Organic acids	[63]	+	−
5	21.27	C_15_H_12_O_5_	271.0626	271.0616	[M-H]−	Naringenin	−5.16	10	Flavonoids	[61]	+	−
6	21.57	C_10_H_16_O	153.1276	153.1274	[M-H]+	Carveol	−1.37	3	Monoterpenoid alcohols	[64]	+	+
7	22.07	C_27_H_32_O_15_	595.1625	595.1668	[M-H]−	Eriocitrin	7.29	12	Flavonoids	[29]	+	+
8	22.32	C_27_H_30_O_15_	593.1478	593.1512	[M-H]−	Vicenin	5.71	13	Flavonoids	[29]	+	+
9	23.48	C_27_H_34_O_15_	597.1869	597.1825	[M-H]−	3,5-di-*C*-beta-glucopyranosylphoretin	−7.37	11	Flavonoids	[29]	+	+
10	23.58	C_27_H_30_O_14_	577.1535	577.1563	[M-H]−	Rhoifolin	7.35	11	Flavonoids	[29]	+	+
11	23.68	C_28_H_32_O_15_	607.1721	607.1668	[M-H]−	3,6-di-*C*-glucosylacacetin	−8.64	3	Flavonoids	[61]	+	+
12	24.69	C_28_H_34_O_14_	593.1827	593.1876	[M-H]−	Poncirin	8.21	12	Flavonoids	[29]	+	−
13	24.78	C_28_H_32_O_14_	591.1703	591.1719	[M-H]−	Acacetin-6-*C*-neohesperidoside (isomargaritene)	2.75	13	Flavonoids	[29]	+	+
14	25.04	C_15_H_24_O	221.1905	221.19	[M-H]+	Spathulenol	−2.31	4	Terpenes	[65]	+	−
15	25.29	C_10_H_16_O	153.1288	153.1274	[M-H]+	*p*-mentha-1,5-8-ol	−9.26	3	Menthane monoterpenoids	[65]	+	−
16	25.39	C_28_H_32_O_14_	591.1771	591.1719	[M-H]−	Acetin-7-*C*-neohesperidoside (fortunellin)	−8.73	13	Flavonoids	[29]	+	+
17	25.94	C_28_H_32_O_15_	607.1659	607.1668	[M-H]−	3,6-di-*C*-glucosylacacetin	1.55	13	Flavonoids	[61]	+	−
18	26.24	C_28_H_34_O_14_	593.1836	593.1876	[M-H]−	Neoporcirin	6.7	12	Flavonoids	[29]	+	+
19	26.85	C_29_H_50_O_2_	431.3867	431.3843	[M-H]+	Vitamin E	6.37	4	Organic compounds	[62]	+	+

**Table 6:** In vitro sun protection factor (SPF) of peel and pulp extracts of *A. cherimola*, *D. kaki*, *C. oblonga* and *F. margarita*; mean ± SD, *n =* 3. Means not shearing the same letter in both columns for one species are significantly different at *p* ≤ 0.05; Tukey’s post hoc test.

	*Annona cherimola*		*Diospyros kaki*		*Cydonia oblonga*		*Fortunella margarita*	
	Peel	Pulp	Peel	Pulp	Peel	Pulp	Peel	Pulp
5%	1.29 ^d^ ± 0.06	0.18 ^a^ ± 0.03	1.39 ^d^ ± 0.02	0.79 ^b^ ± 0.11	2.87 ^d^ ± 0.22	1.50 ^c^ ± 0.07	7.48 ^c^ ± 0.40	6.41 ^c^ ± 0.77
2.5%	0.80 ^c^ ± 0.05	0.05 ^a^ ± 0.00	0.76 ^ab^ ± 0.16	0.52 ^ab^ ± 0.03	1.39 ^c^ ± 0.07	0.75 ^ab^ ± 0.03	2.85 ^a^ ± 0.03	2.46 ^a^ ± 0.06
1%	0.58 ^b^ ± 0.05	0.04 ^a^ ± 0.05	0.51 ^ac^ ± 0.01	0.25 ^c^ ± 0.01	0.97 ^b^ ± 0.16	0.41 ^a^ ± 0.02	1.81 ^ab^ ± 0.16	1.01 ^b^ ± 0.10

## Data Availability

Data are contained within the article.

## Supplementary Materials

The following supporting information can be downloaded at: https://www.mdpi.com/article/10.3390/molecules29051133/s1, Table S1. The MS/MS spectra recorded for the investigated samples.
