# Supplementary material for: Pulp or Peel? Comparative Analysis of the Phytochemical Content and Selected Cosmetic-Related Properties of Annona cherimola L., Diospyros kaki Thumb., Cydonia oblonga Mill. and Fortunella margarita Swingle Pulp and Peel Extracts
Source: Molecules. 2024 Mar 3;29(5):1133. doi: 10.3390/molecules29051133 (PMC10934837; doi:10.3390/molecules29051133)
Supplement: Supplementary file 1 [file molecules-29-01133-s001.zip › molecules-2891418-supplementary.pdf]

SUPPLEMENTARY FILE

Table S1. The MS/MS spectra recorded for the investigated samples

Name of the compound	MS/MS spectrum
(Z)-chlorogenic acid	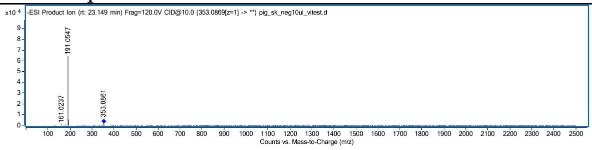
3,5-Di-C-beta-glucopyranosylphoretin	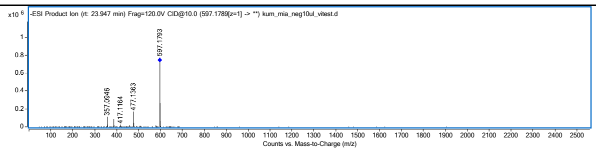
3-beta-glucopyra-nosy-loxy-2-hydroxy-1-(4-hydroxy-3-methophe-nyl)-propan-1-one	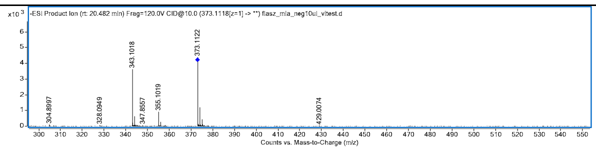
Acetin-7-C-neohesperidoside (fortunellin)	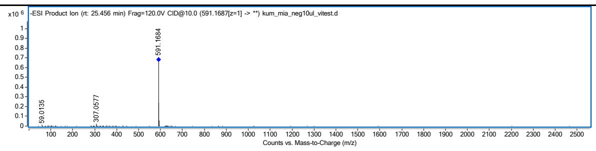
Ascorbyl glucoside	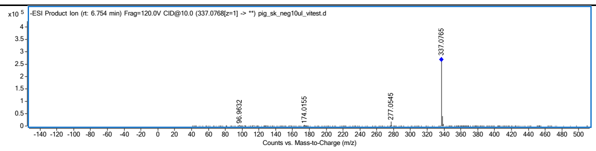
Carveol	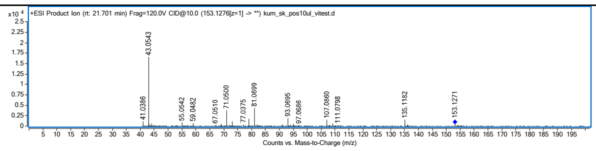
Chlorogenic acid	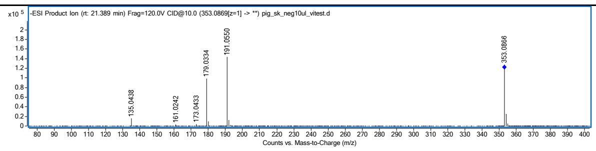

Citric acid	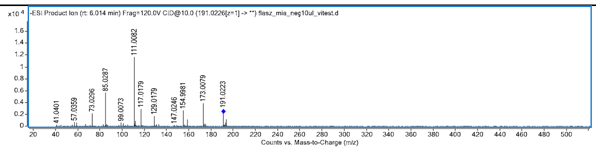
Dehydroascorbic acid	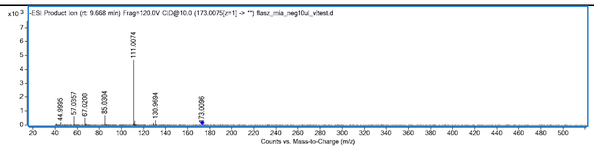
Epicatechin	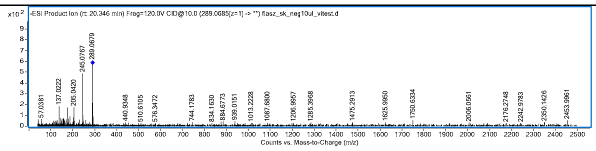
Eriocitrin	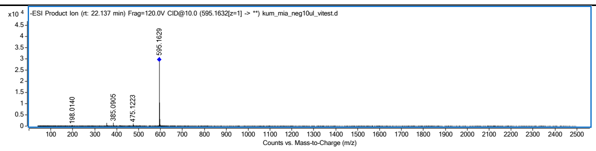
Fructose	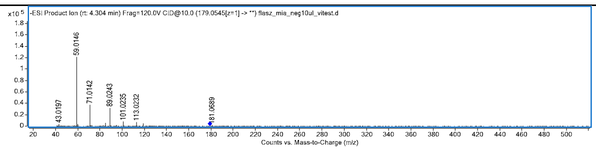
Glucogallin isomer 1	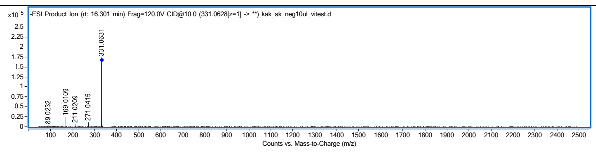
Glucogallin isomer 2	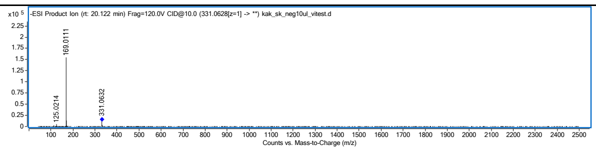
Glucuronic acid	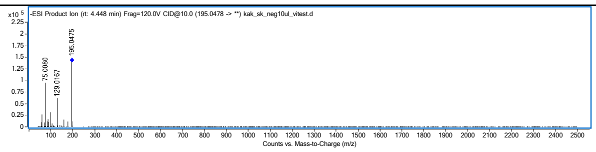

Hexuronic acid	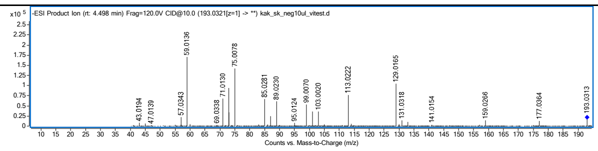
Kaempferol galactoside	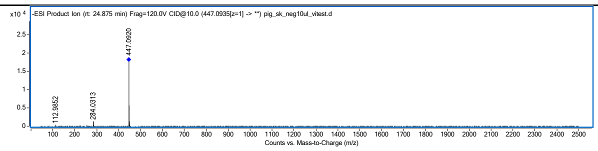
Kaempferol rutinoside	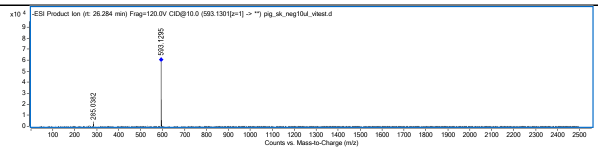
Limocitrol	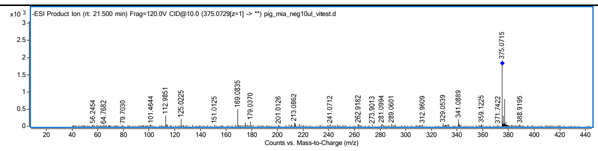
Luteolin glucoside	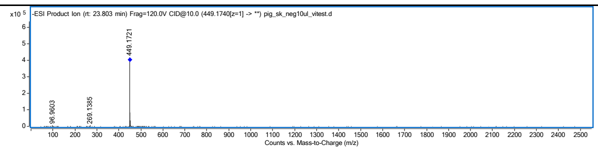
Malic acid	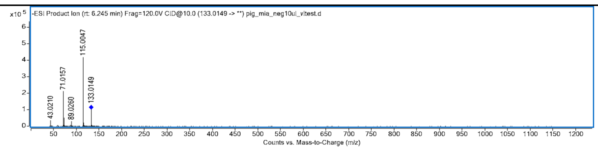
Neochlorogenic acid	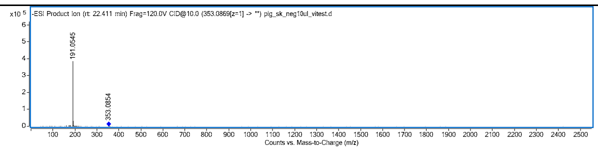
Poncirin	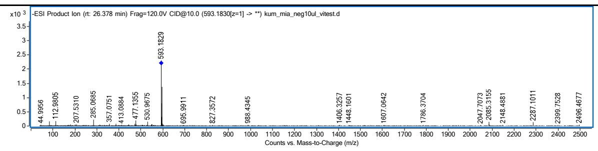

Proline

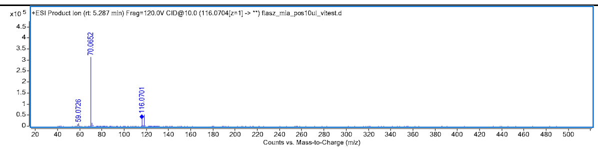

Propylglutaric acid

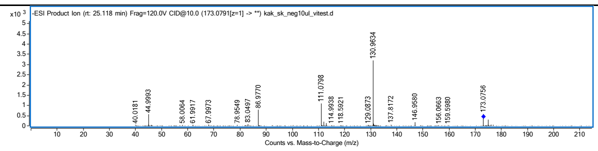

Quercetine galactoside

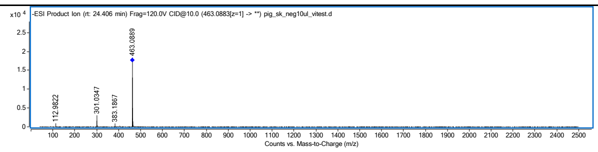

Quinic acid

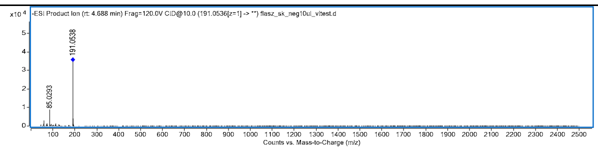

Rhoiflorin

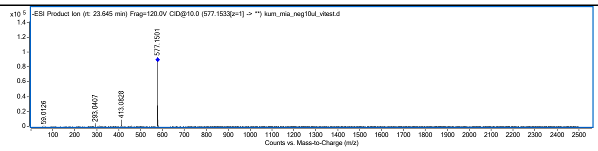

Rutoside

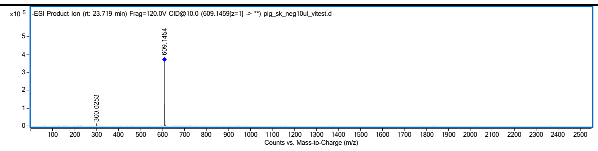

Spathulenol

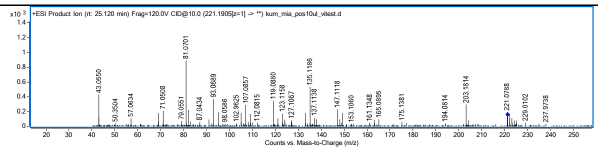

Vicenin

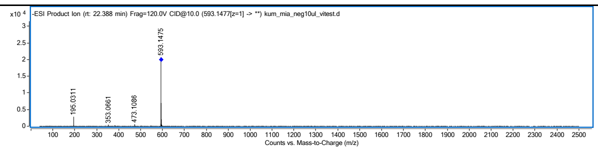

Xylonic acid	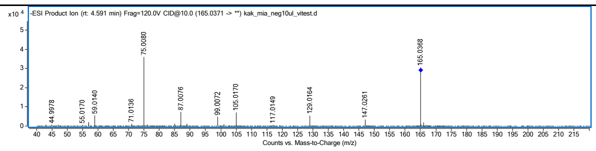
